# Determinants of health-related quality of life in young adults living with perinatally acquired HIV infection in Botswana

**DOI:** 10.4102/sajhivmed.v23i1.1362

**Published:** 2022-04-29

**Authors:** Grace Karugaba, Gloria Thupayagale-Tshweneagae, Mary M. Moleki, Onkabetse V. Mabikwa, Mogomotsi Matshaba

**Affiliations:** 1 Department of Health Studies, University of South Africa, Pretoria, South Africa; 2 Botswana-Baylor Children’s Clinical Centre of Excellence, Gaborone, Botswana; 3 Department of Mathematics and Statistical Sciences, Botswana International University of Science and Technology, Palapye, Botswana; 4 Baylor College of Medicine, Houston, Texas, United States of America

**Keywords:** perinatal HIV, young adulthood, health-related quality of life, ART, Botswana

## Abstract

**Background:**

Due to the successful antiretroviral therapy (ART) programme in Botswana, large numbers of perinatally HIV-infected adolescents are emerging into young adulthood. Young adulthood is a critical period of human development. However, there is lack of information on the factors affecting the health-related quality of life (HRQOL) of young adults living with perinatally acquired HIV (YALPH) in Botswana.

**Objectives:**

The objective of this study was to assess the HRQOL and its determinants among YALPH who were enrolled on ART at Botswana-Baylor Children’s Clinical Centre of Excellence in Gaborone, Botswana.

**Method:**

A cross-sectional study assessed the HRQOL of 509 YALPH aged 18–30 years using the WHOQOL-HIV BREF. Data about other variables of interest were abstracted from medical records. Bivariate analyses were performed using t and Chi-square tests to determine the associations between demographic and clinical variables and general HRQOL. The variables that were associated with the general HRQOL at *P*-value < 0.1 were included in the multivariable analysis using the logistic regression approach.

**Results:**

The majority of participants had good general HRQOL (78.4%). The highest mean HRQOL score was in the Physical domain (5.4 [± 2.9]) and the lowest in the Environment domain (13.8 [± 2.7]). The factors that were significantly associated with the general HRQOL included: level of education attained (*P* = 0.012), employment status (*P* = 0.069), viral load suppression (*P* = 0.073) and self-reported illness (*P* = 0.001).

**Conclusion:**

Interventions that effectively increase educational attainment, employment opportunities, ART adherence, and prevention or management of illness are needed to promote good HRQOL among YALPH in Botswana.

## Introduction

Due to the successful antiretroviral therapy (ART) programme in Botswana, a large number of perinatally HIV-infected adolescents are emerging into young adulthood. Young adulthood (between the ages of 18 and 30 years) is an important period of human development, with long-lasting implications for a person’s economic security, health, and well-being.^1^ During this time, young men and women typically finish school, leave the parental home, find employment, form relationships, start families and pursue those things that help set them on the path to a healthy and productive adult life. However, the presence of a chronic condition such as HIV during this period can interfere with the achievement of the developmental milestones of young adulthood and negatively affect their health-related quality of life (HRQOL).^2^

Research conducted in many settings globally has shown that young adults living with perinatally acquired HIV (YALPH) face several challenges related to growing up with HIV infection, including stigma and discrimination,^3,4,5,6^ problems with long-term adherence to ART and viral suppression,^7,8,9,10^ mental health issues,^5,11,12^ neurocognitive deficits,^5,13,14,15^ difficult choices concerning sexuality and parenthood,^16,17,18^ physical disabilities and impairment,^19^ and settling down after transitioning from paediatric HIV care settings to adult HIV care systems.^5,16,20^ In addition to the HIV-related challenges, YALPH confront challenges similar to those faced by their uninfected peers in Botswana. The Botswana Revised National Youth Policy (2010) identifies the challenges facing the youth, including unemployment, poverty and financial challenges, food insecurity, alcohol and substance use, limited education and training opportunities, limited access to recreational and sports opportunities, and poor access to business opportunities.^21^ These challenges may be risk factors for poor HRQOL in YALPH. Therefore, it is important to understand the HRQOL of YALPH in Botswana, identify protective and risk factors associated with HRQOL, and develop evidence-based interventions to promote their HRQOL.

Quality of life (QOL) is defined by the World Health Organization (WHO) as:

[*A*]n individual’s perception of their position in life in the context of the culture and value systems in which they live and in relation to their goals, expectations, standards, and concerns.^22,23^

Health-related quality of life refers to QOL in the context of health, disease, and treatment. Health-related quality of life is defined as ‘the functional effect of a medical condition and/or its consequent therapy upon a patient’.^22,23^ Health-related quality of life is a multidimensional concept that includes physical, mental, emotional, and social functioning.^24^ Data from HRQOL assessments can be used to inform health policies, guide the allocation of resources, and monitor the effectiveness and quality of care.^22,23^ The investigation of HRQOL among YALPH is very important, because previous research studies of YALPH in other countries found lower HRQOL compared to peers without HIV.^25,26,27^ Therefore, monitoring HRQOL is an important part of providing quality health care to YALPH.

Botswana has made tremendous progress in providing treatment and care services for people living with HIV (PLWH). The Botswana National Anti-Retroviral Therapy Program was established in 2002 with the goal of reducing HIV-related morbidity and mortality and improving the HRQOL of PLWH. The programme provides universal free ART to PLWH under the ‘Treat All’ policy. This is in addition to the diverse psychosocial support programmes offered by the Government and other partners to promote the health and well-being of PLWH. A population-based survey conducted in 2016 showed that the Botswana ART programme was on track to achieving the 2020 UNAIDS 90-90-90 ART and virological suppression targets.^28^ Despite the significant achievements in Botswana’s ART programme in reducing morbidity and mortality among PLWH, little attention has been paid to measuring and monitoring the qualitative outcome of ART and other interventions through HRQOL measurements. Few studies have assessed HRQOL in PLWH in Botswana.^29,30,31^ However, there is no published research on the HRQOL of YALPH, possibly because this is still an emerging population. Consequently, it is unknown what factors affect the HRQOL of YALPH in Botswana and what policies and programmes are needed to enhance their well-being.

The aim of this study was to assess the HRQOL of YALPH and to examine associations between sociodemographic and clinical factors with general HRQOL in order to inform health policy and programming in Botswana.

## Methods

### Study setting, population and sample

This was a cross-sectional study of YALPH who were enrolled on ART at Botswana-Baylor Children’s Clinical Centre of Excellence (Botswana-Baylor) in Gaborone, Botswana, over the period of April 2019 to July 2019. The Botswana-Baylor provides comprehensive clinical and psychosocial support services to youth from a wide geographic area, representing both urban and rural living environments. The patients came to Botswana-Baylor at least once every three months to access a wide range of clinical and psychosocial support services. Thus, collecting data over three months increased the chances of enrolling all qualifying YALPH on ART at the Botswana-Baylor. The WHO recommends a minimum of 300 participants for sites where the WHOQOL-HIV BREF is used for the first time.^23^ Every YALPH arriving at Botswana-Baylor for services on a data collection day was invited to participate in the study until the targeted number of participants was reached. The participants completed the surveys at the clinic while waiting for other services.

### Inclusion criteria

Participants were included if they met the following criteria: (1) documented evidence of perinatal HIV infection in the patient’s medical records maintained at Botswana-Baylor (including documented HIV-positive results, HIV-positive diagnosis in early childhood [0–8 years], having a mother with documented HIV infection and no evidence of other modes of HIV transmission), (2) age (18–30 years), and (3) consent to participate in the study. Young adults living with perinatally acquired HIV who were acutely unwell or had evident cognitive dysfunction or disabilities were deemed incapable of participation and were excluded from the study.

A total of 539 eligible YALPH were invited to participate in the study. Of these, 13 YALPH declined to participate. Nine YALPH refused to participate in the study as they were not able to commit the necessary amount of time and four were not interested in the study. In total, 526 YALPH agreed to participate and have their questionnaire responses linked with their medical records. Seventeen participants were excluded from the analysis because of incomplete forms (< 80% of the questions were completed); thus, we analysed the data from 509 participants.

### Measures and instrument

Health-related quality of life was measured using the WHOQOL-HIV BREF, a shorter version of the WHOQOL**-**HIV, a disease-specific instrument developed and validated by the WHO for measuring HRQOL among PLWH in cross-cultural settings.^22,23,24^ The WHOQOL-HIV BREF has 31 items that assess 30 facets of HRQOL. Each facet is represented by one item. Two items assess overall HRQOL and general health perception.^24,32^ The individual items on the WHOQOL-HIV BREF are rated on a five-point Likert scale ranging from 1 (low or negative perception) to 5 (high or positive perception).^32^ Items that ask about negative perceptions, such as ‘How much do you worry about death?’ are scored using reverse-coding. As a result, higher scores for all items indicate a higher QOL. The WHOQOL-HIV BREF evaluates HRQOL across six domains: Physical, Level of Independence, Psychological, Social Relationships, Environment, and Spirituality. Each domain has a set of facets that represent a description of a state of being, a behaviour, a capacity or potential, or a subjective perception or experience.^24,32^ The average score for each domain is multiplied by four, yielding domain scores ranging from 4 to 20.^24,32^

The Physical domain assesses HIV symptoms, energy and fatigue, pain and discomfort, and sleep and rest. The Psychological domain assesses thinking, learning, memory and concentration, positive feelings, body image and appearance, self-esteem, and negative feelings. The Level of Independence domain assesses dependence on medications or treatments, mobility, activities of daily living, and work capacity. The Social Relationships domain assesses social support, personal relationships, social inclusion, and satisfaction with sex life. The Environment domain assesses physical safety and security, home environment, financial resources, health and social care (accessibility and quality), opportunities for acquiring new information and skills, participation in and opportunities for recreation and leisure activities, physical environment (pollution, noise, traffic, and climate), and transport. Finally, the Spirituality domain assesses forgiveness and blame, concerns about the future, and fear of death and dying.^24,32^ Before this study, the WHOQOL-HIV BREF was used to assess HRQOL in adult PLWH in Botswana by Ndubuka et al.^30^

The following clinical information was abstracted from participants’ electronic medical records maintained at Botswana-Baylor, using a standardised tool: date of birth, ART initiation date, ART regimen, body mass index (BMI kg/m^2^), HIV viral load (copies/mL), and CD4 cell count (cell/mm^3^). The clinical data abstraction was done immediately after the WHOQOL-HIV BREF instrument had been completed. All participants were anonymised and assigned a unique identification number that linked their medical data to the associated WHOQOL-HIV BREF assessment.

### Statistical analysis

Basic statistical analyses were performed to report the mean scores of each HRQOL facet, domain, and the general HRQOL. The WHOQOL-HIV BREF manual for scoring and coding guided the analysis. According to the WHOQOL-HIV BREF manual, the mean scores for each domain range between 4 and 20, with 4 signifying the worst result and 20 signifying the best result. The general HRQOL mean score ranges between 1 and 5, with 1 corresponding to very poor HRQOL and 5 corresponding to very good HRQOL. This study reports the means and standard deviations (s.d.) of all continuous variables and percentages/proportions for categorical variables. Additionally, bivariate analyses were performed using *t* and Chi-square tests to determine the associations between factors of interest and general HRQOL. The variables that were associated with the general HRQOL at *P*-value < 0.1 level of statistical significance were included in the multivariable analysis using the logistic regression approach. The general HRQOL was the outcome of interest and was categorised into a binary variable using 3.0 as the cut-off. A general HRQOL score of less than or equal to 3.0 was considered poor, while a score greater than 3.0 represented good general HRQOL. The predictive ability of the final model was established by reporting scores of a receiver operating characteristic curve.

For triangulation, adjusted factors were also assessed independently against the six domains of HRQOL using the analysis of variance (ANOVA) and *t*-tests. All the analyses were performed using the Statistical Package for Social Sciences (SPSS) version 16.0 (SPSS Inc., Chicago, IL, United States).

### Ethical considerations

Administrative and Ethical approval was received from the Institutional Review Board of Botswana-Baylor Children’s Clinical Centre of Excellence (BBCCOE/14). The study was approved by Botswana’s Ministry of Health and Welfare’s Health Research Development Committee (HRDC) with permit number PPME-13/18/1 Vol VII (318) and University of South Africa, HSHDC/7/012.

## Results

The sociodemographic and clinical characteristics of the sample are presented in Table 1. A total of 509 YALPH on ART participated in the study with a mean age ± s.d. of 21.7 ± 2.6. The proportion of male and female participants was equal (50.0%). The majority of participants were single (98.2%), had lower education (primary or secondary) attainment (71.3%), and lived with their family (90.8%). A large number of participants were unemployed (47.7%). The biological mothers of many participants (62.1%) were alive and on ART, and 14.0% were parents (range 1–3 children) themselves. Regarding sources of information needed in day-to-day living, participants had access to mobile phones (81.5%), television (72.9%), radio (62.6%), newspapers (45.0%) and the Internet (49.3%).

**TABLE 1:** Sociodemographic and clinical characteristics of the sample (*N* = 509).

Social demographic and clinical characteristics	*n*	%	Mean ± s.d.
**Gender**			
Male	254	49.9	
Female	255	50.1	
**Age in years**			21.7 ± 2.6
**Education attainment**			
Lower education	363	71.3	
Upper education	146	28.7	
**Marital status**			
Living as married	6	1.2	
Separated	3	0.6	
Single	500	98.2	
**Living arrangements**			
Family	462	90.8	
Rented	43	8.4	
Boarding school/residential care	4	0.8	
**Employment status**			
Unemployed	243	47.7	
Employed	111	21.8	
In school	155	30.5	
**Parenting**			
Female	63	12.4	
Male	8	1.6	
**Biological mother alive**			
Yes	316	62.1	
No	193	37.9	
**BMI categories (kg/m^2^)**			
< 18.5	196	38.5	
18.5–24.9	273	53.6	
25.0–29.9	35	6.9	
≥ 30	2	0.4	
Missing	3	0.6	
**CD4 counts (cells/mm^3^)**			595 ±356.5
**Viral load (copies/mL)**			
< 400	441	86.6	
> 400	68	13.4	
**Duration on ART (in years)**			12.4 ±4.0
**Feeling ill (self-reported)**			
No	418	82.1	
Yes	91	17.9	
**Self-reported health status**			
Very poor	2	0.4	
Poor	9	1.8	
Neither poor nor good	58	11.4	
Good	240	47.2	
Very good	200	39.3	
**WHO clinical staging**			
Stage 1	90	17.6	
Stage II	73	14.3	
Stage III	191	37.5	
Stage IV	155	30.6	

BMI, body mass index; s.d., standard deviation; ART, antiretroviral therapy; WHO, World Health Organization.

**FIGURE 1:**
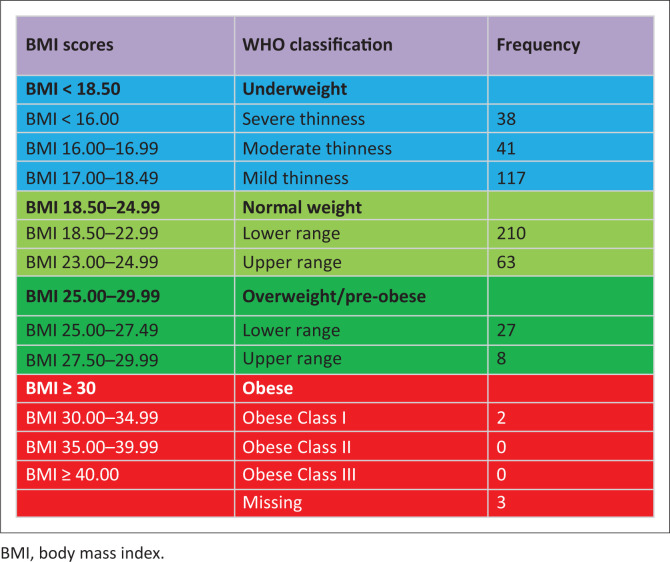
Detailed BMI scores (kg/m^2^).

At the time of the study, all the participants were on ART. The majority of participants (86.6%) had a suppressed viral load (VL < 400 copies/mL) and the mean CD4 count was 595 cells/mm^3^. Only 17.9% of participants were feeling ill (self-reported) and 2.2% had poor health status (self-reported). According to the WHO’s BMI classifications (Figure 1), 38.5% of participants were underweight (BMI: < 18.5 kg/m^2^), with 15.5% being severely thin (BMI: < 17.0 kg/m^2^), and 7.3% were overweight (BMI: ≥ 25.0kg/m^2^). When BMI was categorised further into constituent parts (Figure 1), the majority of participants (64.2%) had a BMI ranging between mild thinness (17.00 kg/m^2^ – 18.49 kg/m^2^) and the lower range of normal (BMI: 18.50 kg/m^2^ – 22.9 kg/m^2^).

### The HRQOL outcomes of participants

#### The mean scores of HRQOL in different facets

**FIGURE 2:**
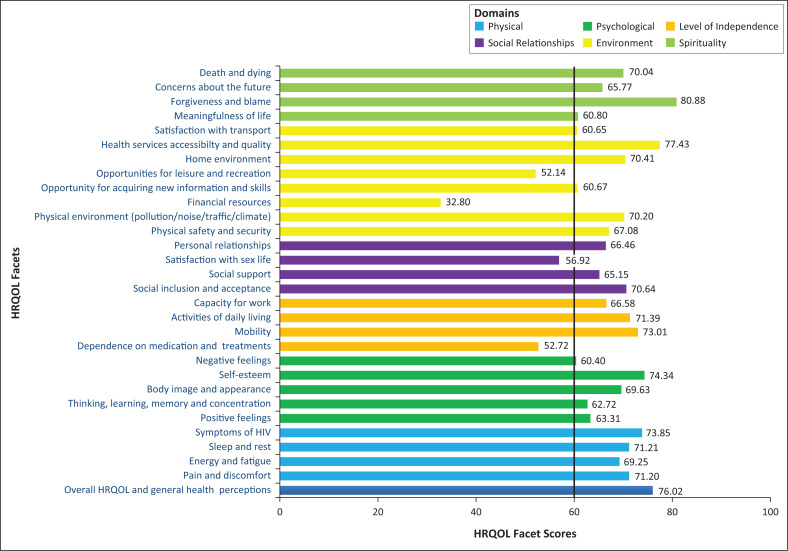
The mean scores of HRQOL in different facets.

The mean HRQOL facet scores in terms of the items on the WHOQOL-HIV BREF are presented in Figure 2. Facet scores were calculated following the WHO’s Users’ Manual for Scoring and Coding WHOQOL-HIV BREF. The scores range from 1 to 5, with 1 indicating very poor QOL and 5 indicating very good QOL. The median score of 3 or weighted median score of 60 was used as the cut-off point to define low or high HRQOL facet scores. Low facet scores were found in the Environment domain (financial resources [32.8], opportunities for leisure and recreation [52.14]), the Level of Independence domain (dependence on medications and treatment [52.72]), and the Social Relationships domain (satisfaction with sex life [56.92]).

#### The mean scores of HRQOL in different domains

Table 2 shows the mean scores for the general HRQOL and the six domains. The majority of participants (78.4%) had good general HRQOL (> 3.0). The mean scores of HRQOL were highest in the Physical domain (15.4) and the lowest in the Environment domain (13.8), followed by the Social Relationships domain (14.4).

**TABLE 2:** Scores for general HRQOL and six domains.

Health-related quality of life measures	*n*	%	Mean ± s.d.
**General HRQOL†**	-	-	3.8 ± 0.7
Physical domain	-	-	15.4 ± 2.9
Psychological domain	-	-	15.0 ± 3.3
Level of Independence domain	-	-	14.6 ± 2.9
Social Relationships domain	-	-	14.4 ± 3.6
Environment domain	-	-	13.8 ± 2.7
Spirituality domain	-	-	15.1 ± 3.1
**General HRQOL** ‡	-	-	
Poor	107	21.0	-
Good	399	78.4	-
Missing	3	0.6	-

HRQOL, health-related quality of life; s.d., standard deviation.

† , General HRQOL score is derived from the questionnaire as the mean of question 1 (‘How would you rate your quality of life?’) and question 2 (‘How satisfied are you with your health?’) based on Users’ Manual for Scoring and Coding WHOQOL-HIV BREF by WHO; it ranges from 1 to 5, with 1 corresponding to very poor HRQOL and 5 corresponding to very good HRQOL.

‡ , General HRQOL score of 3 was used as the cut-off point to define poor and good HRQOL.

### Bivariate analyses

Table 3 presents the sociodemographic and clinical characteristics of the participants by general HRQOL (categorised into two groups: poor or good). Overall, 107 participants (21.0%) had poor general HRQOL, and 399 participants (78.4%) had good general HRQOL. The general HRQOL of three participants (0.6%) could not be classified due to missing information.

**TABLE 3:** Sociodemographic and clinical characteristics of the participants by general HRQOL (poor or good).

Sociodemographic and clinical characteristics	General HRQOL				
	Poor (%)	Good (%)	Poor (Mean ± s.d.)	Good (Mean ± s.d.)	*P*
**Gender**			-	-	0.419
Male	19.70	80.30	-	-	
Female	22.60	77.40	-	-	
**Education attainment**			-	-	0.004
Lower education	24.40	75.60	-	-	
Upper education	13.00	87.00	-	-	
**Employment**			-	-	0.052
Unemployed	25.50	77.10	-	-	
Employed	14.80	85.20	-	-	
In school	18.70	81.30	-	-	
**BMI categories (kg/m^2^)**			-	-	0.73
< 18.5	21.90	78.10	-	-	
18.5–24.9	20.30	79.70	-	-	
25.0–29.9	26.50	73.50	-	-	
≥ 30	0.00	100	-	-	
**Viral load (copies/mL)**			-	-	0.073
< 400	19.90	80.10	-	-	
> 400	29.40	70.60	-	-	
**Feeling ill (self-reported)**			-	-	0.001
No	18.30	81.70	-	-	
Yes	34.40	65.60	-		
**Mean age (in years)**	-	-	21.66 ± 2.75	21.74 ± 2.56	0.789
**Mean CD4 count (cells/mm^3^)**	-	-	586.38 ± 256.0	597.37 ± 380.48	0.778
**Mean duration on ART (in years)**	-	-	12.30 ± 3.75	12.49 ± 4.07	0.673

Note: The *P*-values are from the Chi-square tests for categorical variables (gender, highest level of education, employment status, BMI categories, viral load, illness [self-reported]) and two-sample *t*-tests for the continuous variables (age, CD4 cell count, duration on ART).

BMI, body mass index; HRQOL, health-related quality of life; s.d., standard deviation; ART, antiretroviral therapy.

Based on *P* < 0.1, four factors were significantly associated with the general HRQOL (poor or good). These factors include the highest level of education attained (lower or upper education), employment status (employed or unemployed), VL suppression groups (VL < 400 copies/mL or VL > 400 copies/mL), and self-reported illness (yes or no). The results of the bivariate analysis are shown in Table 3.

### Multivariable analysis

The results fitted using multivariable logistic regression suggest increased odds for good general HRQOL among individuals with upper education attainment and those who are employed. Participants with unsuppressed VL (> 400 copies/mL) and those feeling ill (self-reported) were associated with reduced odds ratios (OR) (Table 4). When participants with upper education were compared to those with lower education, the odds for good general HRQOL increased nearly twofold (OR = 1.97, 95% confidence interval [CI] = 1.11–3.48, *P* = 0.020). Those who were employed had an increased OR (OR = 1.73, 95% CI = 0.92–3.23, *P* = 0.086) when compared to the unemployed. Participants with unsuppressed VL (> 400 copies/mL) had a nearly twofold decrease in good general HRQOL (OR = 0.60, 95% CI = 0.33–1.08, *P* = 0.091) compared to those with suppressed VL (< 400 copies/mL). In addition, participants who were ill (self-reported) had lower ORs for good general HRQOL compared to those who were not ill (OR = 0.42, 95% CI = 0.25–0.70, *P* = 0.001) (see Table 4).

**TABLE 4:** The odds ratios (and 95% confidence intervals) of several covariates adjusted for the General Good HRQOL.

Adjusted for	No. of participants	No. of participants with good HRQOL	OR	95% CI	P
**Education attainment**					
Lower education (reference)	363	272	1.00	1.00	
Upper education	146	127	1.97	1.11–3.48	0.02
**Employment status**					
Unemployed (reference)	243	181	1.00	1.00	
Employed	111	92	1.73	0.92–3.23	0.086
In school	155	126	1.19	0.70–2.01	0.518
**Viral load**					
< 400 copies/mL (reference)	441	351	1.00	1.00	
> 400 copies/mL	68	48	0.6	0.33–1.08	0.091
**Feeling ill (self-reported)**					
No (reference)	418	340	1.00	1.00	
Yes	91	59	0.42	0.25–0.70	0.001

OR, odds ratio; CI, confidence interval; HRQOL, health-related quality of life.

### The Goodness-of-Fit statistics

The area under the curve for the receiver operating characteristic curve was 0.65, suggesting a fair predictive ability of the fitted model.

### Assessment of six HRQOL domains against the adjusted factors

Table 5 shows the mean score difference for the Physical, Psychological, Level of Independence, Social Relationships, Environment, and Spirituality domains against adjusted factors in the multivariable model. At least two of the adjusted factors were significantly different against the Psychological, Level of Independence, Social Relationships, Environment, and Spirituality domains. In contrast, the Physical domain only showed significant results (*P* < 0.01) when investigated against self-reported illness. The mean scores for VLs were insignificantly different across the six domains. Detailed results are provided in Table 5.

**TABLE 5:** The mean score difference in six domains of HRQOL according to adjusted factors.

Covariates adjusted for in the multivariable logistic regression	Physical domain		Psychological domain		Level of Independence domain		Social Relationships domain		Environment domain		Spirituality domain	
	Mean ± s.d.	*P*	Mean ± s.d.	*P*	Mean ± s.d.	*P*	Mean ± s.d.	*P*	Mean ± s.d.	*P*	Mean ± s.d.	*P*
Education attainment**												
Lower education	15.30 ± 2.91	0.15	14.74 ± 3.30	0.00	14.26 ± 2.99	0.00	14.10 ± 3.59	0.01	13.48 ± 2.74	0.00	14.94 ± 3.05	0.05
Upper education	15.71 ± 2.92	-	15.65 ± 2.90	-	15.32 ± 2.55	-	15.07 ± 3.29	-	14.68 ± 2.38	-	15.54 ± 3.16	-
Employment status*												
Unemployed	15.21 ± 2.99	0.21	14.67 ± 3.41	0.02	14.46 ± 2.86	0.18	14.17 ± 3.56	0.44	13.46 ± 2.70	0.01	14.56 ± 3.11	0.00
Employed	15.78 ± 3.00	-	15.76 ± 3.07	-	15.02 ± 2.96	-	14.64 ± 3.37	-	14.15 ± 2.84	-	15.83 ± 3.23	-
In school	15.49 ± 2.72	-	14.97 ± 2.93	-	14.41 ± 2.93	-	14.51 ± 3.60	-	14.18 ± 2.50	-	15.47 ± 2.82	-
Viral load**												
< 400	15.50 ± 2.91	0.13	15.03 ± 3.23	0.60	14.59 ± 2.93	0.65	14.43 ± 3.53	0.42	13.88 ± 2.73	0.30	15.15 ± 3.10	0.52
> 400	14.91 ± 2.92	-	14.81 ± 3.15	-	14.42 ± 2.82	-	14.05 ± 3.50	-	13.51 ± 2.41	-	14.89 ± 3.03	-
Feeling ill**												
No	15.68 ± 2.82	0.00	15.27 ± 3.01	0.00	14.76 ± 2.82	0.00	14.84 ± 3.22	0.00	13.95 ± 2.55	0.04	15.34 ± 2.96	0.00
Yes	14.23 ± 3.06	-	13.74 ± 3.81	-	13.70 ± 3.17	-	12.27 ± 4.07	-	13.29 ± 3.22	-	14.05 ± 3.47	-

s.d., standard deviation.

* , *P*-values are from the analysis of variance (ANOVA) test;

** , two-sample *t*-tests.

## Discussion

This study assessed the HRQOL and its determinants among YALPH on ART in Gaborone, Botswana, using the WHOQOL-HIV BREF. The majority (86.6%) had suppressed VL, optimal immune status, and perceived themselves to be in good or very good health (86.5%). The majority of the participants (78.4%) had good general HRQOL. The highest HRQOL scores were produced in the Physical domain, whereas the lowest scores were produced in the Environment domain. Four socio-economic (education level, employment status) and clinical variables (VL suppression, and illness status [self-reported]) were found to have a strong association with HRQOL of YALPH.

The sociodemographic profile of the participants showed that many YALPH were achieving normal milestones commonly associated with young adulthood, including completing school, finding employment, and parenting, all of which may be reassuring. Data on living arrangements showed that the majority of YALPH (90.8%) still lived in their parental homes. As in any youth with chronic illness, delayed leaving of parental homes likely increased the emotional, social, financial, and other support from family members that the YALPH would not be able to obtain on their own.^33,34^ This means that family-centred interventions should be prioritised in promoting the HRQOL of YALPH in Botswana. Furthermore, many YALPH were parents (14.0%). Therefore, promoting the HRQOL of YALPH parents should be prioritised, and two-generation programming that invests in both young parents and their children should be adopted.

The biomedical and health outcomes among the participants were good, confirming the findings of a population-based survey conducted in 2016 which showed that the Botswana ART programme had progressed towards achieving the UNAIDS 90-90-90 ART and virological suppression goals.^28^ However, despite good health outcomes, many (38.5%) YALPH were underweight. This study found no association between BMI and HRQOL, in contrast to other studies that found a significant relationship between low BMI and reduced HRQOL.^35,36^ The lack of association between BMI and HRQOL found in this study could be attributed to response shift,^37,38^ implying that, over time, YALPH had adapted to their weight and height and had no problems with it. This is supported by a high HRQOL mean score on the facet that measures satisfaction with body image and appearance (69.63). However, generally, being underweight in young adults is a risk factor for ill-health, poor physical functioning, and dissatisfaction with body image.^39,40,41^ Low BMI in perinatally HIV-infected youth could be a result of growth failure (a feature of paediatric HIV infection), illness, or food insecurity.^l5,19,42^ The high prevalence of low BMI in the study population indicates that nutrition education and support should form an integral part of HIV care programmes for YALPH in Botswana. Policies regarding nutritional support to vulnerable populations in Botswana should consider these findings.

The finding that the majority of the participants (78.4%) had good general HRQOL is encouraging, given the challenges associated with HIV as a chronic illness. The highest mean scores of HRQOL were in the Physical domain and the lowest in the Environment domain. The findings are consistent with the findings of a study on the HRQOL of PLWH in Botswana by Ndubuka et al., which found the highest mean score in the Physical domain (15.3) and the lowest in the Environment domain (11.9).^30^ The high mean scores of HRQOL in the Physical domain could be as a result of the fact that the majority of participants in our study were clinically stable on ART, resulting in good physical functioning and Level of Independence. In general, the effects of ART on HRQOL include viral suppression, enhanced immune system functioning, and reduction in AIDS-related illnesses.^43^ The findings are in line with other studies that found good HRQOL in adequately treated young people.^3,16,27,44^ The results support the development of optimal HIV/AIDS treatment and care programmes to optimise the HRQOL of YALPH.

Regarding social relationships, the finding that YALPH had a low mean facet score (56.92) in satisfaction with sex life is concerning, given that the formation of romantic and sexual relationships is key to young adult development.^1,2^ Sexual QOL is related to the emotional, relational and physical aspects of sexuality.^45^ In studies conducted in various settings globally, sexual QOL has been found to be a strong determinant of happiness and life satisfaction.^45,46,47^ In YALPH, satisfaction with sex life may be impacted by various factors such as poor functional status, limited access to sexual and reproductive health services, fear of HIV status disclosure to sexual partners, fear of transmitting HIV to others, intimate partner violence, as well as exposure to coerced sex.^16,17,18^ The findings of this study highlight the importance of fully integrating sexual health services into HIV care for young adults. According to Botswana HIV treatment guidelines, healthcare workers should encourage PLWH to talk openly about their sexual lives and support them in maintaining good sexual and reproductive health.^48^ Further research using qualitative methods is needed to investigate the YALPH narratives underlying low satisfaction with sex life in order to inform the development of specific interventions to improve their sexual QOL.

Health-related quality of life was found to be correlated with the level of educational achievement. The results are not surprising because educational attainment is a major predictor of employment, socio-economic status, health and other personal outcomes in adulthood.^16,30,49,50^ According to research conducted in many settings globally, PLWH with a higher level of education have a higher HRQOL, probably due to better access to information about HIV and ART, which leads to better coping attitudes towards the disease.^16,50,51^ Furthermore, those with a higher level of education may have a larger social support network of family, friends, and workmates resulting in good HRQOL in the social domain.^52^ However, many of the YALPH in this study lacked post-secondary or tertiary education qualifications. In YALPH, educational attainment may be impacted by non-cognitive factors such as illness, visual and hearing impairments and poor educational support from family members, as well as HIV-related cognitive deficits.^5,13,16,19^ These findings call for targeted interventions to support YALPH in acquiring higher level education qualifications as a pathway to good HRQOL. The Government of Botswana, through the Ministry of Tertiary Education, Research, Science and Technology, has a programme that sponsors vulnerable youth to tertiary and technical education. Young adults living with perinatally acquired HIV should be informed about educational support programmes and, where possible, provided with adult mentors to guide them in accessing those services.

The findings of the study demonstrated a statistically significant association between employment status and YALPH HRQOL outcomes, which is consistent with previous research that has found that being employed positively affects the HRQOL of PLWH.^30,50^ In general, employment is an important part of people’s daily lives because it provides structure and a social support network, both of which are important aspects of young adult development.^1,2^ Furthermore, employment and income have been linked to the ability to obtain enough nutritious food and to reduce the effects of HIV-related stress, resulting in improved HRQOL.^53^ However, the high number of YALPH who were unemployed (47.7%) and the low financial resources facet, are causes for concern. Unemployment in youth has been associated with low self-esteem, social isolation, depression, and anxiety, which are risk factors for poor HRQOL.^54^ Within the framework of the Revised National Youth Policy, the Government of Botswana provides a wide range of youth development programmes with the goal of increasing youth employment and financial independence.^21^ Therefore, strategies for enhancing HRQOL in YALPH should include robust interventions to increase their access to employment and entrepreneurship opportunities, particularly for those who are out of school and not working.

Participants’ self-assessment of their health was related with their HRQOL, with those who perceived themselves to be ill having poorer HRQOL compared to those who did not. This finding shows that the functioning and well-being of YALPH are strongly linked to the illnesses they experience or perceive, and thus strategies to address those illnesses and illness perceptions may improve HRQOL. According to the WHO, one of the most important factors influencing QOL is health, and an individual’s subjective assessment of their health is an important aspect of their overall QOL.^55^ Good health is key to young adults’ ability to succeed in education, employment, parenting, social relationships, and achieving their life goals.^56^ Therefore, interventions aimed at improving HRQOL in this population should prioritise providing adequate preventive healthcare and treatment in order to promote good health. Healthcare workers should provide YALPH with adequate information on HIV and ART as well as help in understanding their disease state, setting personal health goals, and developing strategies on how to achieve them.

The study also found that YALPH with unsuppressed VL (> 400 copies/mL) had a lower general HRQOL than those with suppressed VL (< 400 copies/mL). Unsuppressed VL was strongly associated with lower HRQOL across all HRQOL domains. The strong relationship found between VL suppression and HRQOL shows that these can be direct proxies of each other and that strategies for VL suppression are likely to improve HRQOL in YALPH. Viral load levels are one of the biological markers of HIV/AIDS disease progression, the potential for transmission of infection and can have an impact on a person’s self-perception of HRQOL. It is well known that strict adherence to ART (≥ 95%) is required to achieve VL suppression.^10^ The high VL suppression rate found in this study (86.6%) is encouraging, considering that multiple studies have reported challenges with ART adherence and lower rates of VL suppression among YALPH.^7,8,9^ However, the low scores on the ‘dependence on medication or treatments’ facet (52.72) are concerning because they indicate that negative attitudes towards reliance on medications and treatments may impact ART adherence, viral suppression, and HRQOL. These findings highlight the need for strong youth-friendly and evidence-based interventions to foster positive attitudes towards ART, increase ART adherence and maximise success in achieving long-term VL suppression in YALPH.^34,57,58^

Contrary to expectation, the level of CD4 cell count was not associated with the YALPHs’ HRQOL, implying that in the studied population, an increase or reduction in CD4 cell count may not result in a higher or lower HRQOL. The lack of a relationship between CD4 cell count and HRQOL could be due to the fact that almost all participants were clinically stable with optimal immune status (mean = 595 cells/mm^3^). These findings support previous research in PLWH that found no relationship between CD4 cell count and HRQOL.^36,50^ This means that policymakers and clinicians cannot rely solely on CD4 cell count trends to predict HRQOL in the studied population of YALPH. In addition to observing CD4 cell count levels, other factors influencing HRQOL, such as social demographic (education levels, employment status) and clinical (VL levels and illness perception), must be considered. To validate these findings, similar studies in larger YALPH samples with more diverse CD4 cell count scores are needed.

Lastly, this study assessed the HRQOL of YALPH who had been on ART for a long time (mean = 12.4 years). Contrary to expectation, the bivariate analysis showed no relationship between the duration on ART and HRQOL. These results are inconsistent with previous research that found a reduction in HRQOL with an increased duration on ART, possibly due to treatment fatigue.^59^ These results were also inconsistent with previous research which showed an increase in HRQOL with an increased duration on ART.^60^ This implies that the promotion of HRQOL in YALPH should be based on other parameters other than the duration on ART.

### Strengths and limitations

This is the first study to assess the HRQOL of YALPH in Botswana. The study advances current research on HRQOL by presenting the sociodemographic, clinical and HRQOL characteristics of a large sample of YALPH enrolled at a single ART site in Botswana. A precursor to improving the HRQOL of YALPH is knowledge of the factors that influence it, upon which appropriate interventions could be developed. However, the study also had some limitations. Due to the cross-sectional nature of the study, causality could not be established. The whole study sample was selected from Botswana-Baylor, where the majority of participants were clinically stable on ART. The clinical composition of participants on ART may vary across the country, thus future research should consider YALPH in unrepresented subpopulations (those who are not on ART, sick, or hospitalised) to better understand their HRQOL. Lastly, one item on the WHOQOL-HIV BREF instrument: ‘how satisfied are you with your sex life?’ made some participants uncomfortable and the question was likely to lead to errors associated with socially desirable responses, because sex is perceived to be a risk factor for HIV transmission. To minimise the response effect, the researchers explained why the question about satisfaction with sex life was included in this study, to provide the participants with trust and comfort to respond to the question and be able to comfortably rank satisfaction with their sex life on the five-point Linkert scale. Nevertheless, the response effect might have persisted in some cases. Despite these limitations, the researchers are confident that the study results contribute to the literature on the health and well-being of the growing population of YALPH in Botswana.

## Conclusion

The majority of YALPH had good HRQOL, with the highest scores in the Physical domain. These findings show that good HRQOL can be achieved in YALPH who are adequately treated. Higher level education and employment were associated with good HRQOL, whereas unsuppressed VL and self-reported illness were associated with poor HRQOL. Therefore, interventions that provide more educational, employment, financial, ART adherence, and other health-related support are needed to promote the HRQOL of YALPH in Botswana. Special attention should be paid to the HRQOL facets that showed low scores among the YALPH, including financial resources, satisfaction with sex life, access to opportunities for leisure and recreation, and dependence on medication and treatments. Addressing those challenges by policymakers and programmers can help YALPH attainment of developmental milestones and good HRQOL. A multidisciplinary and multisectoral approach is needed to promote the HRQOL of YALPH in Botswana.
